# Cellular Mechanical Phenotypes of Drought-Resistant and Drought-Sensitive Rice Species Distinguished by Double-Resonator Piezoelectric Cytometry Biosensors

**DOI:** 10.3390/bios15060334

**Published:** 2025-05-23

**Authors:** Ding Tang, Tiean Zhou, Weisong Pan, Shimei Wang, Muhammad Ahmad Hassan

**Affiliations:** 1 College of Bioscience and Biotechnology, Hunan Agricultural University, Changsha 410128, China; dingtang1td@outlook.com (D.T.); panweisong@hunau.edu.cn (W.P.); 2 Hunan Engineering Technology Research Center for Cell Mechanics and Functional Analysis, Changsha 410128, China; 3 Rice Research Institute, Anhui Academy of Agricultural Science, Hefei 230031, China; wangshimei@aaas.org.cn (S.W.); ahmaduaf93@aaas.org.cn (M.A.H.)

**Keywords:** cellular phenotypes under drought stress, rice cells, double resonator piezoelectric cytometry, cell-generated surface stress, cell viscoelasticity

## Abstract

Various high-throughput screening methods have been developed to explore plant phenotypes, primarily at the organ and whole plant levels. There is a need to develop phenomics methods at the cellular level to narrow down the genotype to phenotype gap. This study used double-resonator piezoelectric cytometry biosensors to capture the dynamic changes in mechanical phenotypes of living cells of two rice species, drought-resistant Lvhan No. 1 and drought-sensitive 6527, under PEG6000 drought stress. In rice cells of Lvhan No. 1 and 6527, mechanomics parameters, including cell-generated surface stress (ΔS) and viscoelastic parameters (G′, G″, G″/G′), were measured and compared under 5–25% PEG6000. Lvhan No. 1 showed larger viscoelastic but smaller surface stress changes with the same concentration of PEG6000. Moreover, Lvhan No. 1 cells showed better wall–plasma membrane–cytoskeleton continuum structure maintaining ability under drought stress, as proven by transient tension stress (ΔS > 0) and linear G′~ΔS, G″~ΔS relations at higher 15–25% PEG6000, but not for 6527 cells. Additionally, two distinct defense and drought resistance mechanisms were identified through dynamic G″/G′ responses: (i) transient hardening followed by softening recovery under weak drought, and (ii) transient softening followed by hardening recovery under strong drought. The abilities of Lvhan No. 1 cells to both recover from transient hardening to softening and to recover from transient softening to hardening are better than those of 6527 cells. Overall, the dynamic mechanomics phenotypic patterns (ΔS, G′, G″, G″/G′, G′~ΔS, G″~ΔS) verified that Lvhan No. 1 has better drought resistance than that of 6527, which is consistent with the field data.

## 1. Introduction

A key goal of biology is to understand phenotypic characteristics and link them to genetic variants. Generally, a phenotype results from the complex interactions between genotype (morphological, developmental, physiological, pathological or biochemical characteristics, phenology, and behavior) and the environmental factors that can be monitored, quantified, and/or visualized by some technical procedures [1].

In modern biology, phenomics is a screening method that studies plant activities in response to genetic mutation or environmental influences. The capacity to characterize phenomes is significantly less advanced than in describing genomes, and phenomics has not yet made as much progress as genomics in recent decades [2]. Therefore, plant phenomics (PP) has been recognized as a bottleneck in exploring the interactions of genomics and the environment on plants, with its lower advancement restricting the progress of smart breeding and precise cultivation [3,4]. This situation has been significantly improved since the development of numerous high-throughput phenomics methods in the last two decades, which are mainly based on non-destructive and non-invasive imaging techniques for morphological/structural characterizations, sensors, and remote techniques for physiological monitoring (water potential, biochemical compositions (chlorophyll, etc.) [5]. However, it is still a big challenge for phenotype measurements and quantifications to keep pace with and make the best use of the ever-increasing amount of genomic data [6]. In particular, plant phenomics of multiple genomic expression states with countless possibilities of environmental conditions should be measured at a specific time during the development of environmental stress or preferably in a dynamic manner [7].

Within the plant breeding community, it has become the consensus for prospective breeding programs to achieve the following goals: (i) be more efficient (e.g., early discarding of unsuitable material), (ii) have shorter breeding cycles, and (iii) be more productive, increasing the probability of success at the end of the breeding process [8]. Therefore, developing powerful techniques that can characterize and quantify the inherent/internal phenotypes of various genotypes and external phenotypes under various environmental conditions and perturbations is desirable. In this regard, various “omics” techniques such as genomics, transcriptomics, proteomics, and metabolomics have been used but have failed to precisely predict the phenotypes under development and various environmental stresses [9,10]. As the basic unit of organisms, cells are the ideal and logical selection to serve as the key intermediate phenotype connecting the genotype and phenotype of the plants. Indeed, it is strongly suggested that genotyping and external phenotyping should be linked to cellular and tissue-level physiology [11].

Recent research studies have re-examined the key role of micro phenotypes at the cellular and tissue level, emphasizing their significance in bridging the gap between the complex macro phenotype and the genotype [12]. Another challenging question is the mechanism, i.e., how the plant perceives a change in its environment, particularly the first and initial response at the molecular scale, and how this initial response affects the downstream responses across different dimensions [13]. It is well known that the cell wall–plasma membrane–cytoskeleton interface acts as the key player in stress perception, sensing, and signaling to other cellular sections; however, we have limited knowledge about this interface and do not know enough about the key molecular players involved in perceiving the stress [6].

Cell mechanics is closely related to cellular structure and functioning; therefore, it was proposed to quantify cell structure and function based on cytomechanical measurements of cell-generated forces and their viscoelasticity [14]. Cell mechanics is considered the latest and most potent phenotypic screening method in drug screening as it directly targets cell function [15]. Recently, we developed double-resonator piezoelectric cytometry (DRPC) which can measure both the cells-generated traction force and viscoelasticity in a dynamic, real-time, and non-invasive way [14]. The DRPC was successfully applied for the study of the cell adhesion of human umbilical vein endothelial cells [14], identifying different ways of cell death, including apoptosis and pyroptosis [15], and contractile function of H9C2 cells [16]. More recently, it was extended to compare the mechanical properties of Nippobare rice cells and protoplasts under PEG6000 drought stress [17].

Global climate change aggravates abiotic stresses, as drought, heat, and salt stresses are anticipated to increase significantly in the upcoming years [18]. Drought, one of the most devastating abiotic stresses to affect agricultural production worldwide, causes significant crop yield losses [19]. The frequent occurrence of severe drought events has increased concerns over the global water supply chain, posing a substantial threat to the productivity of food crops such as rice, wheat, etc. [20]. Dissecting the drought resistance (DR) mechanism and designing drought-resistant rice varieties are promising strategies to address the challenge of climate change. Phenomics-assisted genetic dissection and molecular design of DR are progressively being used in rice [21]. The researchers used both above- and underground phenomic platforms and multimodal cameras to record 139,040 image-based traits (i-traits) of the phenotypes of entire plants in response to drought stress throughout the whole growth period of rice. They also found 32,586 drought-responsive quantitative trait loci (QTLs), including 2097 uniquely identified QTLs [22]. A genetic engineering breeding technique was employed to improve drought resistance, and the genes of the water-tolerant and drought-tolerant plant Alternanthera philoxeroides (Mart.) were successfully incorporated into the superior conventional rice variety “6527” through embryo immersion, resulting in the successful cultivation of a new rice variety called “Lvhan No. 1” that is both water-efficient and drought-tolerant [23].

Drought tolerance involves changes in cellular biochemistry, physiology, and morphological cellular adaptions that assist in maintaining cell functioning under harsh drought conditions [24]. These alterations result from intricate structural and functional adaptations made by plants, which might directly or indirectly alter cell size, elasticity, water potential, or turgor pressure. These hydraulic and mechanical indexes at the organ level have been suggested for quantifying species tolerances, but the mechanism is still unclear, with some controversial results [25]. For example, it has been suggested that the bulk modulus of elasticity and variations in osmotic potential at the turgor loss point serve as the primary selection criterion for thoroughly screening several genotypes in wheat breeding programs [26]. However, cell mechanical indexes have yet to be established at the cellular level to evaluate drought stress. How plants perceive stress at the molecular scale has always been a challenging question; the answer is critical and open-ended with regard to the understanding of the downstream responses. An emerging field of research is the plasma membrane and its interface with the cell wall and cytoskeleton, which serves as a pragmatic spot for plants to sense environmental signals and control water and solute transport while also initiating downstream signaling and inducing signaling intermediates, such as abscisic acid (ABA). Little is known about how the cell wall–plasma membrane–cytoskeleton interface functions in stress sensing and signaling and what the key molecular players are in stress sensing. And the understanding of the molecular components involved in sensing water deficit within plants and conveying these cues remains largely speculative [6].

The advantage of plant cellular mechanical phenotype characterization techniques lies in their unique and intermediate role in bridging field breeding and molecular breeding, which can be used to predict drought and other abiotic resistances of crop species and explore the molecular components and structures involved in perceiving stress. Plant cell mechanics involve the measurement of cellular forces (including turgor pressure) and viscoelastic parameters. Atomic force microscopy (AFM) is the most popular method for measuring the mechanical properties of plant cells, and particularly for measuring Young’s modulus of cell walls of single cells and for exploring the heterogeneity of the cell wall [27]. Another important mechanical parameter of plant cells is turgor pressure; there are several methods available for the measurement of cellular turgor pressure, including the conventional methods of pressure probe [28], ball tonometry [29], and AFM [30]. However, these methods have some drawbacks, such as causing damage to cells or the need to exert a certain amount of force on cells and the lack of the ability to monitor continuously in a real-time manner. Moreover, these methods are not applicable for measuring negative turgor pressure, which usually occurs in water-deficient situations, including drought stress. For practical applications such as phenotype assays, massive cells have to be used to obtain accurate and statistically meaningful results. Unfortunately, all the above methods are only suitable for single-cell measurement. Therefore, it is conceivable to have some technique that allows for measuring the cytomechanical properties of living plant cells suitable for cellular mechanical phenotype characterization under various stresses with the following features: (1) non-invasive and continuous; (2) capable of measuring both positive turgor pressure (tensile stress) and negative turgor pressure (compressive stress); (3) capable of measuring the viscoelastic parameters at the same time; (4) fast enough to track the early events under stress; (5) suitable for a large number of cells (cell population); and (6) capable of upgrading to high throughput tests. A novel cell mechanics technique, known as the double-resonator piezoelectric cytometry (DRPC) technique, was recently developed by our lab [14,15,16,17] and meets all the above criteria. DRPC allows the real-time, non-invasive monitoring of cells’ generated forces and viscoelasticity from earlier signaling to various cellular functions. It is an ideal cellular mechanical phenotype technique suitable for quantifying changes in the cellular structure and function of living plant cells under various stresses.

The DRPC development was based on a quartz crystal microbalance (QCM), a useful tool for studying complex biomolecular systems at the solution–surface interface [31]. The QCM has also been used to study the cell adhesion and viscoelasticity of animal cells [32]. Our lab was the first to adapt the QCM technique for the study of plant cells based on its viscoelastic sensing [33], and so far we have used it for the study of various abiotic stresses, including low temperature [33], etc. Recently, we applied the DRPC technique for a salt tolerance evaluation of rice varieties [34] and compared the mechanical characteristics of rice cells and protoplasts under PEG6000 drought stress [17].

In this study, the suspension cells of drought-resistant rice variety “Lvhan No. 1” and conventional rice variety “6527” were used as materials, and PEG6000 was used to simulate drought stress; the DRPC was used to monitor the dynamic changes in cell mechanical phenotypic parameters, including surface stress generated and viscoelastic moduli under drought stress, in a real-time manner. This research study aims to explore and discover the differential characteristic fingerprint curves of mechanical phenotypes of rice varieties with different degrees of drought resistance under drought stress to verify the feasibility of the DRPC technique in evaluating drought resistance at the cellular level.

## 2. Materials and Methods

### 2.1. Experimental Materials

Two varieties of rice (*Oryza sativa* L.), the drought-tolerant and water-efficient Lvhan NO. 1 and the high-quality conventional indica rice variety 6527 seeds, were used in the experiments [23]; both were provided by the Rice Research Institute, Anhui Academy of Agricultural Sciences. According to the method of [35], the two rice suspension cell lines were prepared after the calli of these two rice varieties of seeds had been induced.

### 2.2. Reagents and Instruments

The MS medium was purchased from Phyto-Tech, Lenexa, KS, USA. L-Proline (L-Pro), 2,4-dichloro phenoxy acetic acid (2,4-dichlorophenoxyacetic acid), and L-glutamate (L-Glu) were from Shanghai Source Leaf., Shanghai, China. PEG6000 (Polyethylene glycol 6000) was purchased from Wuxi Yatai United Chemical Co., Ltd., Yixing, Jiangsu, China. The 20% Poly Dimethyl Diallyl Ammonium Chloride (PDADMAC) reagent was purchased from Sigma Company, Beijing, China. Anhydrous ethanol was purchased from Sinopharm Chemical Reagent Co., Ltd., Shanghai, China. Ultrapure nitrogen (N_2_) originated from Changsha Xinxiang Gas Analysis Instrument Co., Changsha, China. The high-temperature sterilization pot was purchased from Gaoyi Ruilian Technology Co., Ltd. (Wuhan, Hubei, China). Millipore Milli-Q pure water/ultra-pure water integrated system was purchased from Belimei, Wuhan, Hubei, China. The 250B Crystal Network Analyzer was purchased from Saunders & Associates, Yantai, Shandong, China. The DL-120 ultrasonic cleaning instrument was purchased from Jiekang Company, Jiangmen, Guangdong, China. The 9 MHz AT and BT cut quartz crystal blank chips were manufactured by Hangzhou Zhongjing Electronic Technology Co., Ltd., Hangzhou, Zhejiang, China with gold coating deposited by Beijing Chenjing Electronics Co., Ltd., Beijing, China. The Lumascope720 fluorescence microscope was purchased from Etaluma, Inc., Carlsbad, CA, USA. MMM Climacell environmental plant growth chamber is native to Germany. Countess TM Automated Cell Counter was purchased from Invitrogen, Carlsbad, CA, USA. ME104E/02 Electronic balance was purchased from Mittler-Toledo International Trading (Shanghai) Co., Ltd., Shanghai, China. ZWYR-240 shaker was purchased from Shanghai Zhicheng Analytical Instrument Manufacturing Co., Shanghai, China. PHSJ-3F Ray Magnetic pH meter was purchased from Electric Scientific Instrument Co., Ltd., Shanghai, China; low speed centrifuge was manufactured by Hersey Instruments, Changsha, Hunan, China

### 2.3. Experimental Method

#### 2.3.1. Acquisition of Rice Cells

Rice suspension cells grown for 3–5 days were selected and operated on an ultra-clean table, filtered using a 100 mesh cell screen, and collected with a 500 mesh cell screen. Using the MS (MS salt (4.44 g/L) + sucrose (30 g/L) + L-Pro (0.5 g/L) + L-Glu (0.5 g/L) + CH (0.6 g/L) + 2,4-D (0.2 mg/L)) media, the cells on the 500 mesh cell sieve were rinsed into a beaker, resuspended, and centrifuged twice, when the cell size was almost identical. The cell number was counted, and the above experimental instruments were sterilized at a high temperature in advance.

#### 2.3.2. Cleaning and Surface Modification

Teflon pool cleaning: Teflon wells were put into a beaker with anhydrous ethanol and cleaned for 6–8 min in an ultrasonic bath, and then the liquid was changed to ultra-pure water for ultrasonic cleaning for 6–8 min, and finally the wells were blown dry with ultra-pure N_2_. The above-cleaned wells were put into an ultra-clean work table and sterilized under an ultraviolet lamp for about 15–20 min.

Gold electrode surface cleaning: First, 1 (H_2_O_2_):3 (H_2_SO_4_) Piranha solution was prepared in a beaker and heated in a bath maintained at 80 °C for 20 min. A drop of the heated Piranha solution was put on the gold electrode for more than 30 s, then washed with deionized water, rinsed with anhydrous ethanol, and blown dry with ultrapure N_2_, and the above steps were repeated 3 times. The surface of the cleaned chip was covered with absolute ethanol for 3 min, rinsed with deionized water, and blow-dried with ultrapure N_2_.

Gold electrode surface modification: The above cleaned gold electrode surface was modified with polycation PDADMAC, specifically with 1% PDADMAC modification solution filtered with 0.22 μm microporous membrane, under a dark environment for over 30 min. After modification, the modification solution was buffered with deionized water and dried with ultrapure N_2_ to complete the modification.

#### 2.3.3. Monitoring of the Changes in Surface Stress and Viscoelasticity Generated by Rice Cells Under PEG6000 Stress

The total volume used in each Teflon well was 600 µL. Briefly, 600 µL of rice MS media was added to each of the assembled Teflon wells placed in a MMM Climacell environmental plant growth chamber maintained at 25 °C and 75% RH; the 250B crystal network analyzer was used to monitor the resonance frequency F and dynamic resistance R of the 9 MHz AT and BT chips in real time. Data collection continued for about 3 h, until the F and R data became constant. Then, 300 µL of media was removed and replaced with 300 µL media containing about 5 × 10^4^ rice cells, and the F and R data were collected for about 3 h until the cells reached stable adhesion. Finally, 300 µL of media from the upper fluid of the Teflon well was removed, and 300 µL of equal volume of PEG6000 mother liquor prepared in rice MS media was added to give the final PEG6000 volume percentages of 5%, 10%, 15%, 20%, and 25%; F and R changes were continuously monitored in real time with the 250B crystal network analyzer.

#### 2.3.4. DRPC Principle and Calculation Formulas for Cellular Mechanical Parameters

The theoretical part of DRPC was described in detail in our previous publication [14]. Here, we only briefly introduce its principle and the equations for cell-generated surface stress and viscoelasticity.

The DRPC chips are made of silicon dioxide quartz crystals, which are anisotropic in that their physical properties are different along different molecular axes of the crystal. A slice of blank quartz used for making a resonator was obtained by cutting the crystal at specific angles to each axis. The two most popular cuts, AT and BT, adopted in DRPC, with rotation cut angles of 35°15′ and −49°, respectively, determine their sensitivities to mechanical parameters of mass, viscoelasticity, and lateral stress of the coupled load exerted on the crystals.

When the quartz crystal chips are used as the substrates for monitoring cell adhesion, the frequency change induced during cell adhesion is mainly affected by the surface stress exerted on the quartz crystal ΔS, cell mass, and cell viscoelasticity. The total relative frequency shift caused by the cell adhesion process is expressed as

Δf/f_0_ = Δfs/f_0_ + Δfm/f_0_ + Δf_visco_/f_0_ (1)

Here, Δf is the total frequency shift, f_0_ is the frequency of AT and BT cut crystals, and Δf_s_, Δf_m_, and Δf_visco_ are frequency shifts contributed by cell-generated surface stress ΔS, cell mass Δm, and cell viscoelasticity, respectively.

It has previously been demonstrated that AT and BT cuts have similar frequency sensitivities to mass but with different response slopes; it was assumed that they have similar frequency sensitivities to viscoelasticity, as proved by using PEG2000 (Sinopharm Chemical Reagent Co., Ltd., Shanghai, China) solutions [14]. Therefore, the living cells would produce the same trends of frequency shifts for both AT and BT cut crystals, but with different magnitudes. However, the frequency–stress coefficient was almost the same for AT and BT cut quartz crystals, but with opposite signs. The exerted lateral stress would change the frequency determining third- and higher-order elastic constants in a cut angle-dependent way [36].

From the known theoretical equations characterizing the mass, viscoelasticity, and lateral stress-induced frequency shifts in AT and BT cut crystals and under the assumed conditions that AT cut and BT cut crystals have the same frequency and surface status modified with the same adhesion molecule or material in the same way, the following equation characterizing the dynamic surface stress generated by cells’ ΔS_t_ at any given time t during their adhesion was derived by Zhou et al. [14].

ΔS_t_ = f_0_^−1^(K^AT^ − K^BT^)^−1^(Δf_t_^AT^t_q_^AT^ − Δf_t_^BT^t_q_^BT^) (2)

In Formula (2), the stress coefficients for AT and BT cuts are constants: K^AT^ = 2.75 × 10^−12^ cm^2^ dyn^−1^, K^BT^ = −2.65 × 10^−12^ cm^2^ dyn^−1^. f_0_^AT^ = f_0_^BT^ = f_0_ is the resonance frequency of the quartz crystal. t_q_^AT^ and t_q_^BT^ are the thicknesses of the AT and BT cut crystals, respectively, determined by their frequency constants N^AT^, N^BT^, and their frequencies, N^AT^ = 1.661 MHz·mm = 0.1661 MHz·cm, N^BT^ = 2.536 MHz·mm = 0.2536 MHz·cm. Therefore, thickness t_q_ can also be determined for a specific frequency. The specific expressions for AT and BT cut crystals are t_q_^AT^ = 0.1661/f_0_^AT^ and t_q_^BT^ = 0.2536/f_0_^BT^. Therefore, in Formula (2), only Δf_t_^AT^ and Δf_t_^BT^ are variable frequency shifts in AT cut and BT cut crystals at specific time t, contrasting their reference frequency shifts, for example, stable values in cells’ free culture media.

When f_0_^AT^ = f_0_^BT^ = 9 MHz, t_q_^AT^ = 0.0185 cm, t_q_^BT^ = 0.0282 cm.

Formula (2) can be simplified to

ΔS = 380.7Δf^AT^ − 580.4Δf^BT^ (3)

The unit for Δf^AT^, Δf^BT^ is Hz, and the unit for surface stress ΔS is dyne/cm.

It should be indicated that Equations (2) and (3) are derived under the conditions of dynamic changes in cell mass and viscoelasticity and generated surface stress, but the cells’ culture media are maintained unchanged. However, during abiotic stresses, the density, viscosity, and viscoelasticity of the media would change depending on the type of stress. For drought stress, as simulated by PEG6000 solutions, the viscoelasticity of the media would change. However, Equations (2) and (3) are still valid after the conditions of changing culture media were considered [17]. As long as the AT cut crystal has the same frequency and surface state and is modified with the same molecule or material in the same way as the BT cut crystal, the precise measurement of the surface stress ΔS (traction force or turgor pressure) produced by the cells on the crystal can be guaranteed, which is universal. When ΔS is negative, it indicates that the surface stress produced by the cells themselves is a compressive stress, and the cells are shrinking; when ΔS is positive, the surface stress produced by the cells themselves is a tension stress; that is, the cells are in an expanding state (i.e., turgor pressure). Once ΔS has been obtained, frequency shifts contributed by the viscoelasticity of the cells can be obtained by subtracting the surface stress induced frequency change from the total frequency shifts. The following equations can be used to calculate surface stress-induced frequency shifts for 9 MHz crystals:

Δf_s_^AT^(Hz) = f_0_^AT^K^AT^ΔS/t_q_^AT^ = 9 × 10^6^ × 2.75 × 10^−12^ × ΔS/0.0185 = 0.001338ΔS (4)

Δf_s_^BT^(Hz) = f_0_^BT^K^BT^ΔS/t_q_^BT^ = 9 × 10^6^ × (−2.65 × 10^−12^) × ΔS/0.0282 = −0.0008457ΔS (5)

The rest frequency shift is obtained from the total frequency shift minus the surface stress-induced one:

Δf_cells_ (Hz) = ΔF − Δf_s_ (6)

As the frequency shift caused by cell mass is minor and can be ignored, the above equation-calculated frequency shift is mainly contributed by the cell’s viscoelasticity.

Because the MHz shear wave’s penetration depth to the cells is much smaller than that of the cells themselves, cells can be considered as a semi-infinite viscoelastic load. The storage and loss moduli of the cells are calculated by the following two equations [14]:

G′ = π^2^Z_q_^2^(ρ_c_f_0_^2^)^−1^(ΔR^2^/16π^2^L_q_^2^ − Δf^2^) (7)

G″ = −π^2^Z_q_^2^ΔfΔR/2ρ_c_L_q_f_0_^2^ (8)

ρ_c_ is the density of the cells assumed to be the same as pure water 1.00 g/cm^3^, and Z_q_, L_q_ are the acoustic impedance and motional inductance of the resonator in cell culture media. Δf and ΔR are changes in frequency and motional resistance induced by cells relative to air. In reality, we measured cell adhesion-induced changes in frequency and motional resistance relative to cell culture media.

Δf = Δf_cells_ + Δf_media_ = (f_cells_ − f_media_) + (f_media_ − f_air_) (9)

ΔR = ΔR_cells_ + ΔR_media_ = (R_cells_ − R_media_) + (R_media_ − R_air_) (10)

The frequency and motional resistance of quartz resonators in media relative to air can be considered the same as in pure water at the measured temperature, which can be theoretically calculated [14]. Eventually, we obtained the following four formulas characterizing the cells’ viscoelasticity at 25 °C for 9 MHz AT and BT cut crystals.

G′^AT^(pascal) = 9.576 × 10^−3^((ΔR^AT^)^^2^/16π^2^(L_q_^AT^)^^2^ − (Δf^AT^)^^2^) (11)

G″^AT^(pascal) = −1.525 × 10^−3^Δf^AT^ΔR^AT^/L_q_^AT^ (12)

G′^BT^(pascal) = 2.233 × 10^−2^ ((ΔR^BT^)^^2^/16π^2^(L_q_^BT^)^^2^ − (Δf^BT^)^^2^) (13)

G″^BT^(pascal) = −3.556 × 10^−3^Δf^BT^ΔR^BT^/L_q_^BT^ (14)

#### 2.3.5. Data Analysis

Data analysis and mapping were performed using Origin 2018 and Excel (Microsoft Office 365).

**Figure 1:**
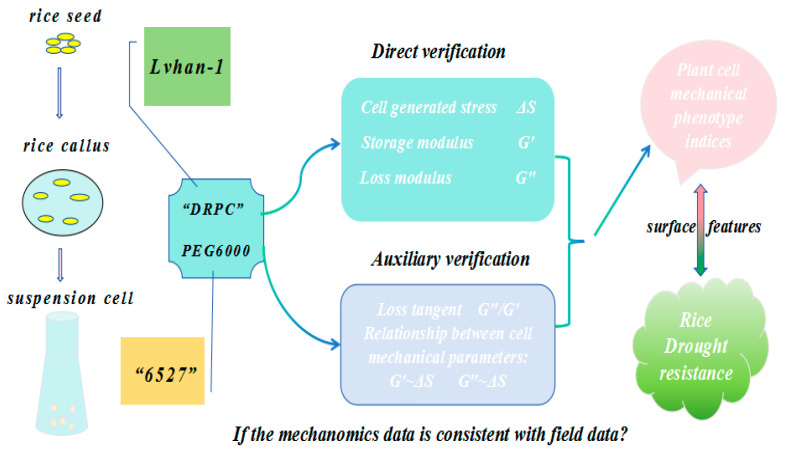
Schematic illustration of a typical DRPC application in a drought stress study.

Therefore, the DRPC technique allows direct measurement of cell-generated surface stress ΔS and viscoelastic moduli G′, G″ in a dynamic manner, and the auxiliary parameter loss tangent G″/G′ and relations between cell-generated forces and viscoelasticity can also be obtained and analyzed. The purpose of this work is to apply DRPC to distinguish rice cells originating from two different species of known relative drought resistances under the treatments of PEG6000 stress to verify whether the dynamic mechanomics phenotype patterns or changes in mechanical phenotype indices are consistent with the field data (Figure 1).

## 3. Results

### 3.1. Cell Adhesion Morphology

**Figure 2:**
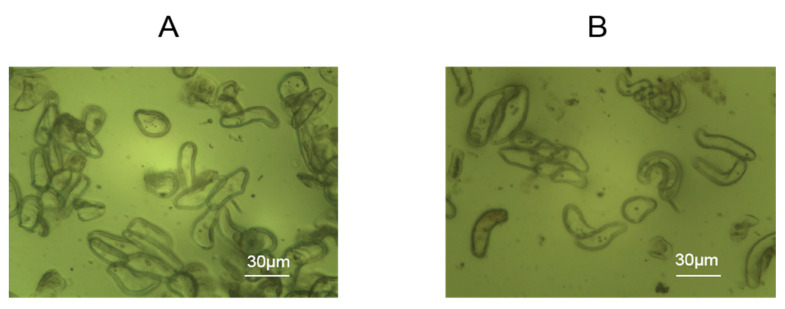
Morphologies of rice cells attached to PDADMAC-modified gold electrodes of DRPC chips. (**A**) Lvhan No. 1, (**B**) 6527.

Rice cells were added to the assembled wells, containing chips electrostatically modified with 1% PDADMAC to allow for cell adhesion for 24 h. As shown in Figure 2, through the effect of electrostatic modification, the cells adhered well to the modified gold electrodes with rectangle morphologies reflecting the effects of the cell wall.

### 3.2. Lvhan No. 1 and 6527 Cells-Generated Surface Stresses Under PEG6000 Stress

After adding 5 × 10^4^ Lvhan No. 1 or 6527 rice cells to 9 MHz AT and BT cut chips, there were general trends of frequency drop and resistance increase, indicating the successful adhesion of the cells to the chips (Figures S1 and S2). The adhered cells of both Lvhan No. 1 and 6527 were then subjected to treatments of 5%, 10%, 15%, 20%, and 25% PEG6000 simulated drought stress; the DRPC traces showed a sudden drop in F and rising R for all the PEG6000 concentrations tested, indicating enhanced adhesion due to contraction of the cells, and the degrees of F and R changes increased with the increase in PEG6000 concentration for both AT and BT cut crystals and both Lvhan No. 1 and 6527 rice cells (Figures S1 and S2). Following these initial sudden changes in both F and R, reversed changes in both F and R were observed except for 5% PEG6000. At that concentration, Lvhan No. 1 cells showed a continuous but slight increase in R and a decrease in F, and the rebound degrees increased with the increase in PEG6000 concentrations.

**Figure 3:**
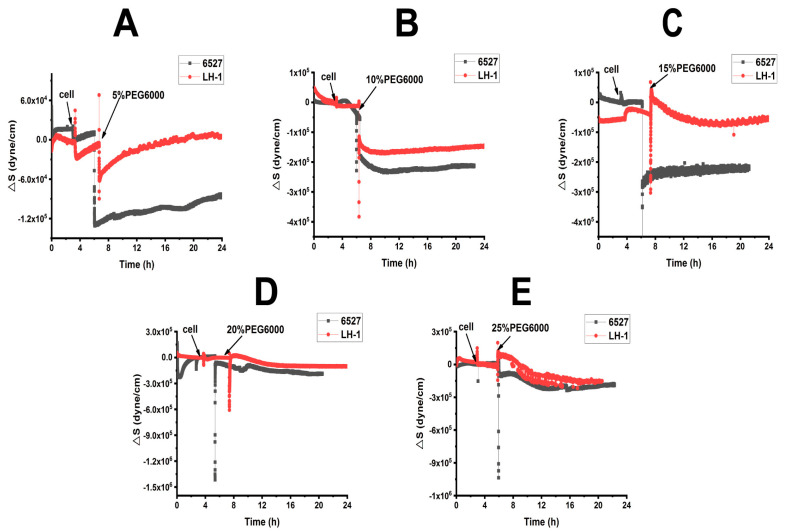
Cells-generated surface stresses of Lvhan No. 1 and 6527 rice cells during their adhesions to 9 MHz AT and BT cut chips, followed by the treatments of different concentrations of PEG6000 stress by the DRPC technique. (**A**): 5%, (**B**): 10%, (**C**): 15%, (**D**): 20%, (**E**): 25%.

**Figure 4:**
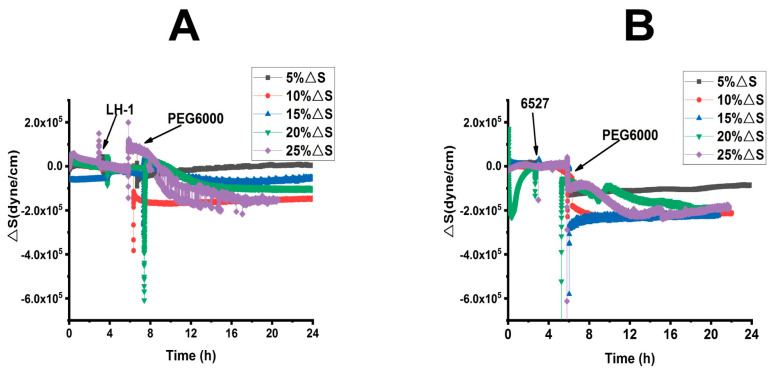
Superimposed time-dependent response curves of surface stress generated by Lvhan No. 1 and 6527 rice cells during their adhesions followed by treatments of different concentrations of PEG6000 captured by 9 MHz DRPC. (**A**): Lvhan No. 1, (**B**): 6527.

From Figures S1 and S2, we calculated the surface stress ΔS generated by Lvhan No. 1 and 6527 rice cells under the treatments of 5%, 10%, 15%, 20% and 25% PEG6000, which are presented in Figure 3 and Figure 4. At lower PEG6000, both types of cells mainly generated compressive stress due to contraction. At 5% PEG6000, after a steep drop, ΔS reversed to increase, and the reverse degree of Lvhan No. 1 was more significant than that of 6527. On the other hand, at 10% PEG6000, ΔS became relatively stable after the initial sudden drop. At both 5% and 10% PEG6000 concentrations, the changes in ΔS for Lvhan No. 1 were much smaller than those of 6527, implying higher drought resistance for Lvhan No. 1. Meanwhile, beginning at 15% PEG6000, the ΔS response of Lvhan No. 1 changed the pattern from the initial contractile stress to tensile stress (ΔS > 0); this brief increase in ΔS possibly reflects the Hechtian strands-related protection mechanism, as the contraction of protoplasts would stretch the Hechtian strands, resulting in tensile stress [37]. Still, this phenomenon was not observed in 6527’s ΔS response, which always generated initial contractile stress, although a slight recovery at 15% PEG6000 or reversed direction at the late stage of 20% and 25% PEG6000 appeared. However, they never exceeded 0, i.e., maintaining their contractile state. Again, at each PEG6000 concentration, the change in ΔS was smaller for Lvhan No. 1 than 6527, indicating better drought resistance.

When 15% PEG6000 was added, the surface stress ΔS of Lvhan No. 1 rice cells experienced a sharp increase and then dropped rapidly, followed by a rebound tension stress. This complicated profile possibly reflects the initial stretch of Hechtian strands, followed by the de-polymerization and re-assembly of the cytoskeleton structure. At this concentration, the Lvhan No. 1 rice cells could still repair themselves after being damaged by PEG6000 stress. When Lvhan No. 1 rice cells were treated with a high concentration of 20% PEG6000 stress, surface stress ΔS in the late stage slightly recovered but in an overall flat state, indicating that the Hechtian strands connecting the cell wall and protoplasts were partially damaged and Lvhan No. 1 cells reached their maximum drought endurance point and they may need a much longer time to recover. The ΔS profile at this concentration is similar to 6527 at 10% PEG6000. When Lvhan No. 1 rice cells were treated with an even higher concentration of 25%PEG6000 stress, ΔS experienced an initial sudden increase then decreased monotonically, presenting a “comb” state without recovery, indicating irreversible cellular structure damage.

When 15% PEG6000 was injected into the living environment of 6527 cells, the cells generated a cliff-like contractile stress. At this concentration, the Hechtian strands were probably broken. For 6527 rice cells subjected to 20% and 25% PEG6000 stress, overall, the cells generated monotonous contractile stress without recovery; most of the cells could not repair themselves and probably lost their activity. In summary, the maximum drought stress can be tolerated by Lvhan No. 1 and 6527, which were found to be 20% and 10% PEG6000, respectively, once again indicating better drought resistance for Lvhan No. 1 cells.

Combined with Figure 3 and Figure 4, for plant cells subjected to drought resistance, the changes in cells generated surface stress ΔS indicated that the cells would experience initial elastic change at a lower concentration of PEG6000, then gradually transformed to plastic change. The PEG6000 concentration at which ΔS changed from elastic to plastic depends on the rice species; the higher the PEG6000 concentration (Lvhan No. 1), the better the drought resistance. At lower PEG6000 concentration, the smaller the ΔS, the better the drought resistance of the rice species (Lvhan No. 1). In summary, Lvhan No. 1 cells showed consistently better drought resistance with the field results, which also indicates that the cells-generated surface stress ΔS can be used to characterize stress resistance and can become a new mechanical index for the identification of drought-resistant cultivars.

### 3.3. Changes in Viscoelastic Moduli of Lvhan No. 1 and 6527 Cells Under PEG6000 Stress

**Figure 5:**
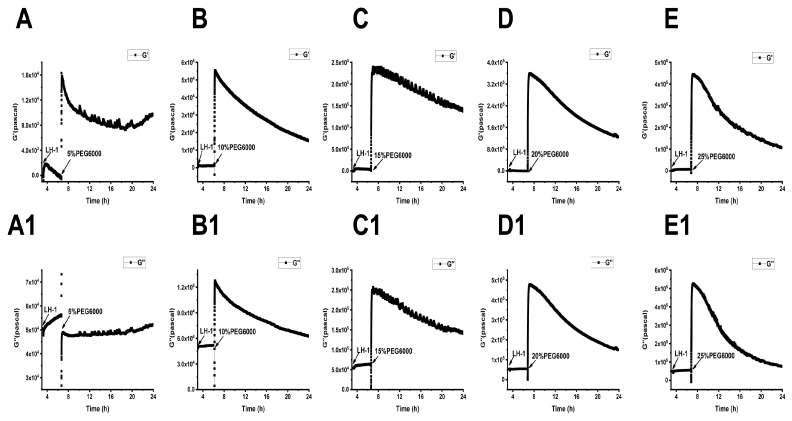
Dynamic changes in viscoelastic moduli of Lvhan No. 1 cells during their adhesions followed by the treatments of different concentrations of PEG6000 monitored by 9 MHz DRPC. (**A**–**E**): Storage modulus G′, (**A1**–**E1**): loss modulus G″. (**A**,**A1**): 5%PEG6000, (**B**,**B1**): 10%PEG6000, (**C**,**C1**): 15%PEG6000, (**D**,**D1**): 20%PEG6000, (**E**,**E1**): 25%PEG6000.

**Figure 6:**
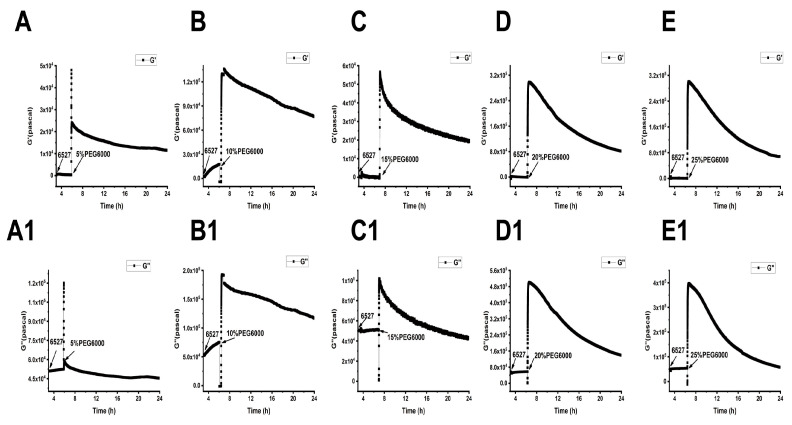
Dynamic changes in viscoelastic moduli of 6527 cells during their adhesions followed by the treatments of different concentrations of PEG6000 monitored by 9 MHz DRPC. (**A**–**E**): Storage modulus G′, (**A1**–**E1**): loss modulus G″. (**A**,**A1**): 5%PEG6000, (**B**,**B1**): 10%PEG6000, (**C**,**C1**): 15%PEG6000, (**D**,**D1**): 20%PEG6000, (**E**,**E1**): 25%PEG6000.

**Figure 7:**
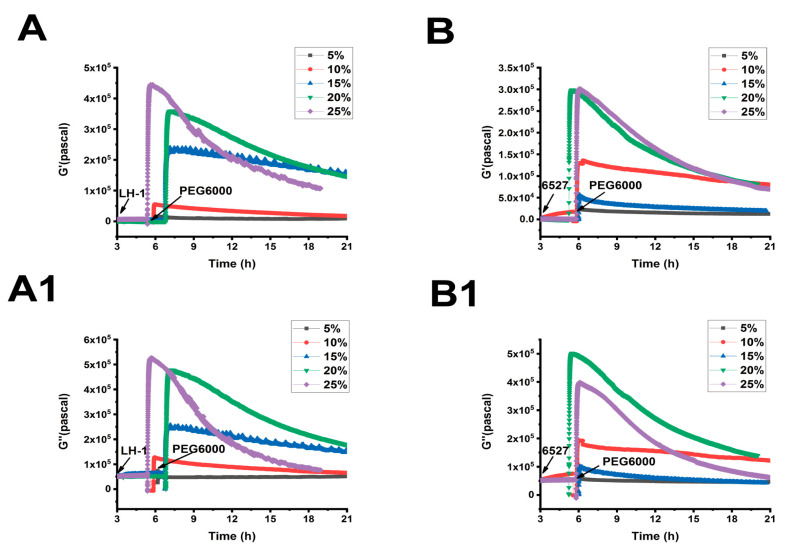
Superimposed time-dependent viscoelastic response curves of Lvhan No. 1 and 6527 rice cells under the stress of different concentrations of PEG6000 captured by 9 MHz DRPC. (**A**,**A1**): Lvhan No. 1, (**B**,**B1**): 6527, (**A**,**B**): storage modulus G′, (**A1**,**B1**): loss modulus G″.

As shown in Figure 5, Figure 6 and Figure 7, we can observe the dynamic changes in viscoelastic moduli in different rice varieties after data comparison and analysis, and the change trends of moduli also reflect the structural change in the cell wall. In general, the viscoelastic moduli of the two rice cells increased transiently, then they decreased after adding PEG6000, and the maximum values initially reached in both G′ and G″ increased with the increase in PEG6000 concentration. After reaching their extreme values, the decay rates of the moduli also increased with the increase in PEG6000 concentration. The changes in viscoelastic moduli of Lvhan No. 1 cells followed this pattern exactly, indicating that the cells of Lvhan No. 1 had a wide linear adaptive range of viscoelastic changes under drought stress. However, the viscoelastic moduli of 6527 cells at 15% PEG6000 were less than those at 10% PEG6000, corresponding to the cliff-like drop in the surface stress generated by 6527 cells at 15% PEG6000 (Figure 3C). In addition, the G′ dynamic response curve of 6527 cells at 25% PEG6000 almost overlapped with that at 20% PEG6000 (Figure 7B). The loss modulus G″ at 25% PEG6000 was even smaller than that at 20% PEG6000. These results suggest that the adjustable viscoelastic changes of 6527 cells under drought stress are not as wide as those of Lvhan No. 1, which may reflect that the stability of its Hechtian strands and/or the cell wall–plasma membrane–cytoskeletal continuum is not as good as that of Lvhan No. 1. Overall, through comparing the dynamic change trends of the G′, G″ moduli of these two varieties of rice cells under different concentration of PEG6000 stress, it could be verified that the drought resistance of Lvhan No. 1 cells is better than that of 6527.

**Figure 8:**
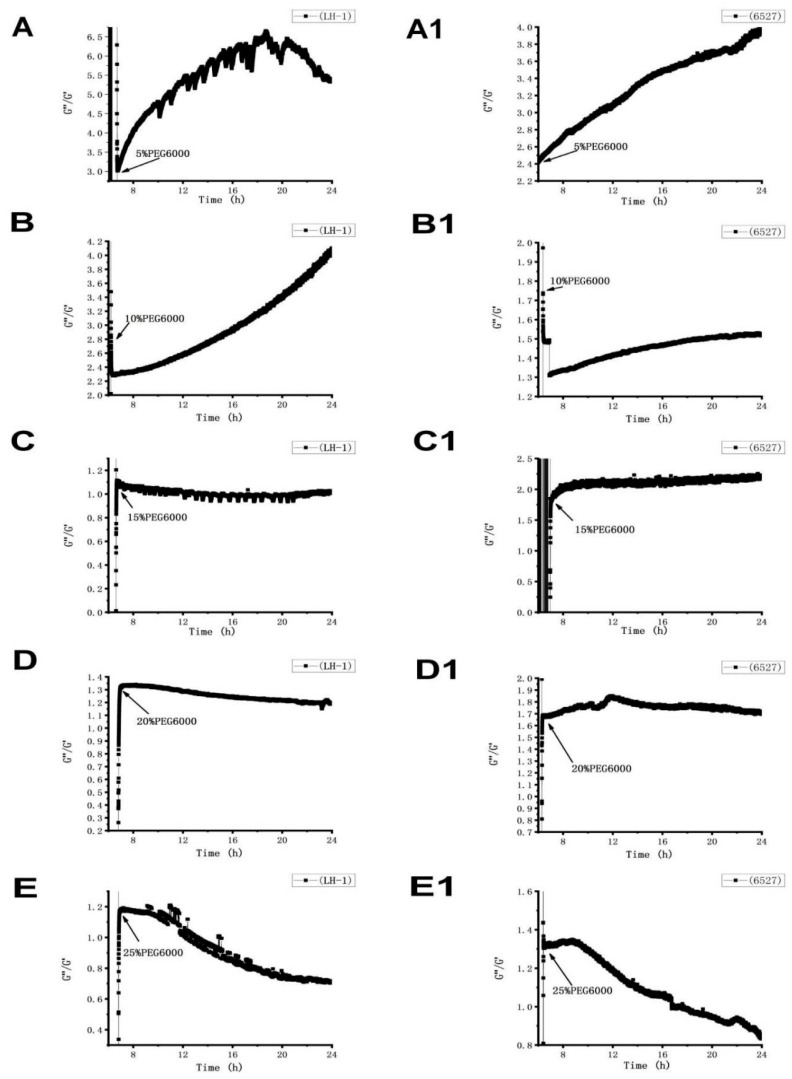
Time-dependent loss tangent G″/G′ change curves of Lvhan No. 1 and 6527 rice cells under the stress of different concentrations of PEG6000. (**A**–**E**): Lvhan No. 1, (**A1**–**E1**): 6527.

The loss tangent of Lvhan No. 1 and 6527 cells showed consistent time-dependent changes under the stress of different PEG6000 concentrations (Figure 8). According to the different concentrations of PEG6000, it can be divided into two regions. At the lower concentrations of 5% and 10%, the G″/G′ of Lvhan No. 1 and 6527 cells first experienced a transient drop and then gradually increased, indicating that the cells first activated the emergency protection mechanism to harden instantly and then slowly softened toward the previous more liquid-like state before stress. The change in loss tangent under 5% PEG6000 stress was greater than that of under 10% PEG6000 stress, and the degree of softening of Lvhan No. 1 cells was greater than that of 6527 under the same PEG6000 concentration. When the PEG6000 concentration was equal to or greater than 15%, the G″/G′ of both Lvhan No. 1 and 6527 cells first demonstrated a transient and instant increase and then gradually decreased, indicating that the cells first activated the emergency protection mechanism to soften the cells, and then slowly hardened toward the previous more solid-like state before stress. The recovery degree of cell hardening increased with the increase in PEG6000 concentration, and the final G″/G′ value of Lvhan No. 1 was less than 6527 at the same PEG6000 concentration; that is, the recovery degree of hardening of Lvhan No. 1 cells was greater than that of 6527. Therefore, the dynamic change curves of the loss tangent of plant cells under different concentrations of PEG6000 stress effectively demonstrate the intelligent defense mechanism of plant cells, that is, under low-intensity stress, when the cells themselves have sufficient strength, the emergency defense mode of intense response is adopted; and in the case of high-intensity stress, to avoid irreversible damage to the cells themselves, the cells would first adopt the emergency defense mechanism of softening. The latter defense mechanism may involve plasmolysis, the pulling of Hechtian strands. While the two rice cells are compared, it can be concluded that the abilities of Lvhan No. 1 cells to both recover to softening after transient hardening and to recover hardening after transient softening are better than those of 6527 cells, further proving that the drought resistance of Lvhan No. 1 cells is greater than 6527.

**Figure 9:**
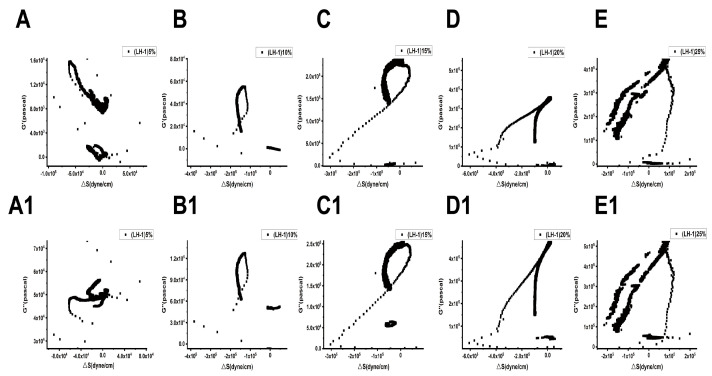
Relations between cell-generated forces and viscoelastic moduli of Lvhan No. 1 rice cells under the treatments of different concentrations of PEG6000. (**A**–**E**): G′~ΔS, (**A1**–**E1**): G″~ΔS. (**A**,**A1**): 5%PEG6000, (**B**,**B1**): 10%PEG6000, (**C**,**C1**): 15%PEG6000, (**D**,**D1**): 20%PEG6000, (**E**,**E1**): 25%PEG6000.

**Figure 10:**
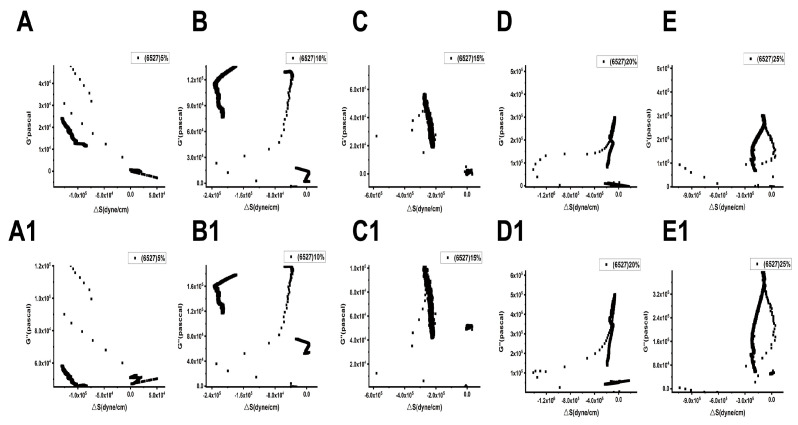
Relations between cell-generated forces and viscoelastic moduli of 6527 rice cells under the treatments of different concentrations of PEG6000. (**A**–**E**): G′~ΔS, (**A1**–**E1**): G″~ΔS. (**A**,**A1**): 5%PEG6000, (**B**,**B1**): 10%PEG6000, (**C**,**C1**): 15%PEG6000, (**D**,**D1**): 20%PEG6000, (**E**,**E1**): 25%PEG6000.

Figure 9 and Figure 10 show the relationships between the surface stress ΔS generated and the viscoelastic moduli (G′, G″) of Lvhan No. 1 and 6527 cells under different concentrations of PEG6000 stress, respectively. The results showed that for Lvhan No. 1 cells, there were complex nonlinear relationships between ΔS and G′, G″; but there were linear relationships only in some concentrations and limited periods. Under the lower concentrations of 5% and 10% PEG6000 stress, ΔS showed nonlinear relationships with G′ and G″. When the PEG6000 concentration was equal to or greater than 15%, the ΔS of the Lvhan No. 1 cells showed linear relationships with G′ and G′ in some time regions. The 15–25% PEG6000 concentrations corresponded to the tensile stress generated by Lvhan No. 1 cells due to the stretch of the Hechtian strands (Figure 3C,E and Figure 9); here, we confirmed experimentally that the increase in the tensile stress of plant cells is proportional to the viscoelastic moduli of the plant cells during the period when the tensile stress is dominant, which is consistent with the theoretical prediction of tensegrity model proposed in animal cells [38]; that is, the mechanical behavior in living plant cells under the dominance of tensile stress is also consistent with the tensegrity model. In such situations, the cell wall–plasma membrane–cytoskeleton forms a relatively complete cell continuum structure, which helps to resist drought and other stresses. There were no apparent relationships between ΔS and G′, G″ in 6527 rice cells under 5–25% PEG6000 drought stress, which was related to the fact that we did not observe tensile stress in our experiments where the cells could be irreversibly damaged due to the structural instability of the Hechtian strands under PEG6000 stress. By analyzing the relationships between ΔS and G′, G″ in Lvhan No. 1 and 6527 rice cells under PEG6000 stress, we concluded that the structural integrity of the Lvhan No. 1 rice cells is better than that of the 6527 cells, therefore showing better drought resistance.

## 4. Discussion

### 4.1. Evaluation of Crop Resistance at the Cellular Level

Recently, crop resistance breeding has been a pragmatic approach to improving crop yield under different biotic and abiotic conditions. Modern gene editing and genetic engineering technologies have provided new insight into ongoing crop breeding practices [3,39]. The success of any crop breeding practice can be evaluated in terms of field phenotype and final yield. With the rapid increase and development in genomic data, it not only requires extensive land area, sufficient labor resources, and enough time to test all the genomic traits in the field, but often requires more than 10 years to breed a new crop variety [40]. Crop breeding is quite similar to the discovery of new drugs. The drug discovery industry frequently uses molecular and cellular pre-screening techniques to speed up new drug discovery and reduce the number of drug candidates before proceeding into clinical trials; this can be carried out by evaluating the efficacy and toxicity of potential drug candidates as well as whether they are permitted to proceed to the final clinical trials by employing targeted molecular interactions and cellular functional phenotypic tests. Here, we proposed monitoring functional and phenotypic alterations of crop cells under biological and abiotic stresses to quantitatively assess crop varieties’ resistance results, thereby speeding up the variety selection for resistance and significantly reducing the number of gene-associated varieties that eventually enter field trials [22].

Monitoring plant cell and physiological function indicators provides a possibility for the quantitative prediction of crop variety phenotype and resistance results [41]. Tissue culture methods have been used to improve stress tolerance in plants [42]. Under different stress conditions, the structure and function of the cell wall, plasma membrane, cytoskeleton, and other organelles of plant cells underwent dynamic changes [43]. In principle, if the dynamic changes in different structures and functions of plant cells are monitored in a real-time manner, it can quantitatively evaluate the resistance and damage degree of different cell structures under various stresses and provide novel ideas and targets for breeding crop-resistant varieties [17]. At present, optical techniques and technologies such as atomic force microscopy (AFM) for testing cell mechanical property, markers for live imaging of the plant cytoskeleton have been used for research and the quantitative determination of cell structures and functions [44]. However, most of these techniques and instruments are expensive, unable to achieve rapid and non-destructive dynamic monitoring, require reagent labeling, and are inconvenient to operate, so they have not been accurately used for crop resistance and cell-level assessments [9,45]. In this study, the double-resonator piezoelectric cytometry (DRPC) technique was used to monitor the dynamic changes in cellular forces and viscoelasticity of rice cells with varying resistance to drought stress in a real-time manner to find and establish the fingerprint characteristic curve of the mechanophenotype of cells under early drought stresses. Thus, a new matrix of indicators for drought resistance based on cytomechanical phenotype was preliminarily established.

### 4.2. Drought Resistance Evaluation Indexes

Crop drought assessment indexes are the tools that were used to quantify the responses of crop plants to drought stress and the magnitude of drought-induced damage to crop plants [46]. These indexes cover a wide range of aspects, from physiology and biochemistry to morphology and yield [47,48]. They are used to evaluate the level of drought resistance and the impacts on crops. The following are commonly used crop drought assessment indexes: (A) physiological indexes that include relative water content (RWC), leaf water potential, leaf turgor pressure loss point, and stomatal conductance; (B) biochemical indexes that include osmoregulatory substances, antioxidant enzymatic activities, and reactive oxygen species (ROS) levels; (C) morphological indexes that include the degree of leaf wilting, root characteristics, and plant biomass; (D) photosynthetic indexes that include photosynthetic rate, chlorophyll contents, and fluorescence parameters; (E) yield-related indexes that include yield (grain yield, biological yield) and harvest index; (F) molecular and gene expression indexes that include changes in gene expression levels and protein expression profiles under drought stress; and (G) comprehensive assessment indexes that include drought resistance index and drought sensitivity index [47,48].

Traditional physiological and biochemical indexes indirectly exhibit the response to drought stress, and it is very challenging to directly correlate these responses to the cellular structural damage mechanism [49]. The morphological indexes are the core content of plant phenotypic research and discovery [50]. Plant cells may have obvious morphological changes before the visible deformation of roots and leaves, and changes in gene and protein expression levels will also reflect changes in plant cells’ overall performance. Therefore, cell-level assessment is expected to be a sensitive and effective method for comprehensively evaluating crop drought resistance [51]. Current evaluation indexes and methods at the cellular level include (a) cell membrane stability (conductivity method, Trypan blue staining method), (b) cell wall composition analysis (cellulose/lignin content, Fourier infrared spectroscopy), (c) organelle damage (chloroplast ultrastructure observation, mitochondrial membrane potential), and (d) osmotic regulation ability (cell turgor pressure, osmotic potential).

The indexes mentioned above directly depict the damage and adaptability of different cellular structures under drought stress. However, most currently used measurement methods rely on destructive sampling and lack non-destructive continuous quantitative monitoring methods. In this study, double resonator piezoelectric cytometry (DRPC) was used to monitor the changes in the cellular mechanical phenotypes of two rice varieties (Lvhan No. 1 and 6527: both varieties known to have significant differences in their drought resistance capacity in the field) at different concentrations of PEG6000. DRPC technology is currently the only cell mechanics technology that can quantitatively measure the cellular force and viscoelasticity of a large number of cells (cell population) at the same time, and its sub-second to second-level sampling capability allows it to obtain the same or even faster mechanical response signals than earlier biochemical signals such as calcium ions, reactive oxygen species, pH, plant hormones, and metabolites. Its non-destructive, label-free, and long-term monitoring characteristics enable it to track the damage and adaptation processes of cells subsequent to the initial fast response to drought stress and to explore the drought resistance mechanism. It is expected that the DRPC technique, which can quantitatively characterize cellular structure and function, would obtain more complete and accurate cellular phenotype information under drought stress than single or even multiple biochemical signals or other cell-level indicators and provide better predictive assessment for drought resistance. The distinctive and time-dependent mechanical curves captured by DRPC reflect the dynamic changes in the cell wall, plasma membrane, cytoskeleton, cell wall–plasma membrane–cytoskeleton continuum structure, and cellular forces or turgor pressure. Based on the experimental results, Table 1 exhibits and summarizes the parameters of cellular mechanical phenotypes, their significance for drought resistance, and their possible association with cell structures.

### 4.3. Limitations and Future Developments

This experimental study compared the dynamic response curves of cellular force (ΔS) and viscoelastic modulus (G′, G″ and G″/G′) generated by two rice varieties with varying drought tolerance capacity under the drought stress of different concentrations of PEG6000 by using the DRPC technique. The results exhibited that (1) at the same PEG6000 concentration, more negative contraction stress ΔS closely correlates with cellular shrinkage, leading to poor drought tolerance. Additionally, the surface stress ΔS generated by cells is proportional to the turgor pressure of cells. Under the same PEG6000 concentration, the smaller the change in ΔS, the stronger the turgor pressure maintenance ability and the better the drought resistance of cells. (2) The viscoelastic moduli of cells directly reflect the relationship between stress and strain under external forces, especially the storage modulus (which is the ratio of stress and strain) directly characterized the cell resistance.

Cell storage modulus = cell resistance = stress strength (intensity of stress)/strain (stress-induced change) = applied stress/induced deformability

Greater storage modulus G′ means that more stress (stress strength) is required to produce per-unit strain (stress-induced change); therefore, the larger the G′, the greater the capacity of cellular resistance [14]. Our results showed that under the same concentration of PEG6000, the change in ΔS of Lvhan No. 1 cells was smaller than that of 6527, but the viscoelastic moduli G′ and G″ of Lvhan No. 1 cells were larger than those of 6527. The results of both cellular force and viscoelastic moduli showed that the drought resistance of Lvhan No. 1 was better than 6527, which was not only consistent with the plant stress physiology theory but also with the results of drought stress experiments in the field and at the seedling stage. The feasibility of DRPC for evaluating the drought resistance of rice varieties was proven. In this study, it was observed that Lvhan No. 1 cells produced transient tensile stress under a high concentration of PEG6000, while 6527 cells primarily produced negative compressive stress. This difference was attributed to the normal functioning or loss of functioning of the Hechtian strands. Under drought stress, not only the cell storage modulus G′ increase, but the loss modulus G″ also increases, so it is difficult to assess the precise change in the stiffness or softness of the cell. Therefore, due to this reason, we used the loss tangent G″/G′ to characterize it. Based on the dynamic change patterns of G″/G′ under drought stress and different concentrations of PEG6000, two different response mechanisms were found in two different types of cells under low and high concentrations of PEG6000: (i) at low concentration, the cells were strong enough and first transiently hardened and then softened; (2) at high concentrations, in order to protect the cells from irreversible damage, the cells first transiently softened and then hardened. Whether for the degree of transient hardening and then softening at low concentrations or the degree of transient softening and then hardening at high concentrations, Lvhan No. 1 is better than 6527, which further indicates that the drought resistance of Lvhan No. 1 is more significant than that of 6527. It has been predicted that plant cells also follow the widely confirmed cellular tensegrity model of animal cells [52], but there are few experimental data to support it. Since plant cells do not have an adherent function like animal cells, no force-sensing molecules similar to animal cell integrins have been discovered and identified yet. Therefore, no linear relationships between the cells-generated forces and the viscoelastic moduli were observed in this study during the non-specific adhesion of plant cells to the positively charged PDADMAC coating, as opposed to what was observed during the adhesion of animal cells on fibronectin and RGD coatings [14]. However, at high concentrations of PEG6000, Lvhan No. 1 showed tensile stress that may be involved in microfilaments. Therefore, linear relationships between the cellular forces ΔS and the viscoelastic moduli G′, G″ were observed for Lvhan No. 1 cells, which were not observed in 6527 cells without tensile stress. Furthermore, Lvhan No. 1 cells maintained a relatively complete cell wall–plasma membrane–cytoskeleton continuum under drought conditions; hence, it was further proved that Lvhan No. 1 had better drought resistance than 6527. The quantification of all these mechanomics parameters (i.e., cells-generated surface stress ΔS, viscoelastic parameters G′, G″, G″/G′) was measured using the DRPC technique. In brief, DRPC has the unique advantages of non-destructive, dynamic, continuous, and simultaneous quantification of cells generated surface stress ΔS and viscoelastic parameters G′, G″, G″ under drought stress. The presented evidence from DRPC results (based on changes in ΔS, G′, and G″) established that Lvhan No. 1 is more drought-resistant than 6527. Furthermore, two different defense and drought resistance mechanisms were found by G″/G′, namely, transient hardening and then soft recovery under weak drought and transient softening and then hard recovery under strong drought. According to G′~ΔS and G″~ΔS, Lvhan No. 1 cells under high-concentration drought stress exhibited linear relationships, which was consistent with the tensegrity model. The above experimental results are consistent with the plant stress physiological theory and model; our interpretation is also consistent with the structural characteristics of plant cells. Therefore, in order to correlate cell mechanical parameters to cell morphology and dynamic changes in different cellular structures, it is the perfect model to realize simultaneous visual observations of cell morphology and cellular structure while measuring cell mechanical parameters at the same time. It is also desirable to measure the biochemical signals during the early events in plant abiotic stress, preferably in a real-time manner, while measuring cell mechanical parameters to correlate changes in cytomechanical parameters to biochemical components. In order to screen and compare the drought resistance of rice cells under different conditions and in different varieties, it is necessary to break through the limitation of the current four-channel measurement instrument. It would be ideal to employ the innovative and high-throughput cell mechanics chip structure that we previously suggested [53].

**Figure 11:**
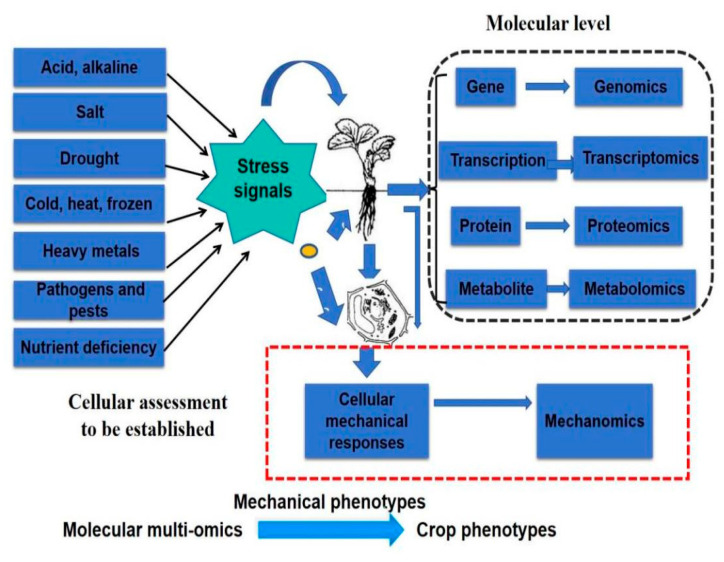
Schematic illustration of cellular mechanical phenotypes to bridge the molecular multi-omics to crop phenotypes under various biotic and abiotic stresses.

The successful development of high-throughput cell mechanics chips and measuring instruments will lay the foundation for the establishment of a cellular mechanomics platform for resistance assessment and breeding of different crops under various biotic and abiotic stresses, as shown in Figure 11.

## 5. Conclusions

Conventional breeding is a longer process and it usually takes about 10 years to release a new cultivar, including rice. By using genetic and molecular techniques, the speed of breeding high-yielding, disease-resistant, stress-resistant crops has been accelerated. However, the prediction accuracy of molecular breeding, including genomic and marker-assisted selection, must be examined through phenotypic data. With the development of various high-throughput phenotyping (HTP) techniques, it is still an issue to take the resources, time, and cost to grow the candidate crops in various environments. Here, we proposed that some of the phenotypic traits of plant populations, like stress tolerance in the field, can be pre-screened using a cell-based assay, specifically cellular mechanical phenotype, to largely reduce the candidate crops for field tests. We used a novel cell mechanics technique, DRPC, recently developed by our lab to capture the dynamic changes in mechanical phenotypes of living cells of two rice species, drought-resistant Lvhan No. 1 and drought-sensitive 6527, under PEG6000 drought stresses. The results indicated that the relative changes in cellular force and viscoelastic parameters captured within 24 h of drought treatment could be used to evaluate the two rice varieties’ relative drought resistance, consistent with the plant stress physiology theory and the known field results. Moreover, the dynamic cytomechanical curves directly reflect the dynamic changes in the cellular structures. An intelligent distinct defense and drought resistance mechanism of rice cells were revealed as transient hardening under weak drought and transient softening under strong drought. The results presented in this work demonstrated the potential to develop a new cellular mechanical phenotype platform to screen for biotic and abiotic stress-resistant crop varieties, as shown in Figure 11.

## Figures and Tables

**Table 1:** Parameters of cellular mechanical phenotypes, significance for drought resistance, and possible relationship with cellular structures.

Parameters of Cellular Mechanical Phenotypes	Drought Resistance Significance	Possible Relationship with Cellular Structures
**Surface stress ΔS generated by cells**	(1) Under normal isotonic conditions, cells are in force balance (mechanical homeostasis), with ΔS near zero (ΔS ≈ 0); under PEG6000 drought stress, cells produce contraction stress, ΔS < 0. Under the same concentration of PEG6000 stress, the more negative ΔS is, the worse the drought resistance is. (2) The higher the PEG6000 concentration at which ΔS produces irreversible plastic changes, the better the drought resistance is. (3) Under high concentration PEG6000 stress, cells produce transient tensile stress (ΔS > 0) and have good drought resistance.	When the PEG6000 concentration exceeds a certain value, ΔS changes monotonically over time and shows no recovery trend, indicating that irreversible plastic stress has occurred in the cell structure. The higher the PEG6000 concentration that produces irreversible plastic stress, the better the drought resistance. If cells produce tensile stress (ΔS > 0) under high concentration PEG6000 stress, it indicates that the structure and function of the Hechtian strands are normal and the drought resistance is good; if ΔS < 0, it indicates that the structure of the Hechtian strands is very weak or has undergone irreversible damage or rupture, and the drought resistance is poor.
**Cell viscoelastic function indexes G′, G″, G″/G′**	(1) Under drought stress, the storage modulus G′ and loss modulus G″ of cells increase. Similarly, under PEG6000 concentration stress, the greater the increase in G′ and G″ (the higher G′ and G″), the better the cell drought resistance. (2) In the entire PEG6000 concentration range, do the maximum values of G′ and G″ increase monotonically with the increase in PEG6000 concentration? If so, the cells show a good linear viscoelastic range and have good cell drought resistance; if not, under high concentrations of PEG6000, the cells cannot maintain a good linear viscoelastic range due to the destruction of cell structural integrity. (3) The larger the G″/G′, the softer the cell, and the smaller the G″/G′, the harder the cell. In the same PEG6000 concentration range, the larger the G″/G′ range, that is, the better the regulation function of the cell to soften and harden, the wider the regulation range, and the better the cell drought resistance.	The cell storage modulus G′ response under drought stress reflects the change in cell wall hardness. G″/G′ reflects the softness and hardness of the cell. The width of the concentration range of G′ and G″ increases monotonically with the increase in PEG6000 concentration, so the larger the adjustable viscoelastic range of the cell, and the better the integrity of the cell structure.
**Cell force and viscoelasticity correlation curves G′~ΔS, G″~ΔS**	At high PEG6000 concentrations, are there linear G′~ΔS, G″~ΔS relationship curves that conform to the cellular tensegrity model? Yes: the cell wall–plasma membrane–cytoskeleton continuum has good structural integrity and good drought resistance.	At high PEG6000 concentrations, the linear G′~ΔS and G″~ΔS relationships that conform to the cellular tensegrity model indicate that the cell wall–plasma membrane–cytoskeleton continuum has good structural integrity.

## Data Availability

Data collected and/or analyzed during this study can be obtained from the corresponding author upon reasonable request.

## Supplementary Materials

The following supporting information can be downloaded at https://www.mdpi.com/article/10.3390/bios15060334/s1, Figure S1: Changes in frequency and motional resistance of 9 MHz AT and BT cut chips during the adhesions of Lvhan No.1 rice cells followed by the treatments of different concentrations of PEG6000 stresses. (A, B, C, D, E): AT cut, (A1, B1, C1, D1, E1): BT cut, (A, A1): 5% PEG6000, (B, B1): 10%PEG6000, (C, C1) 15%PEG6000, (D, D1): 20%PEG6000, (E, E1): 25% PEG6000. Figure S2: Changes in frequency and motional resistance of 9 MHz AT and BT cut chips during the adhesions of 6527 rice cells followed by the treatments of different concentrations of PEG6000 stresses. (A, B, C, D, E): AT cut, (A1, B1, C1, D1, E1): BT cut, (A, A1): 5% PEG6000, (B, B1): 10%PEG6000, (C, C1) 15%PEG6000, (D, D1): 20%PEG6000, (E, E1): 25% PEG6000.
