# Supplementary material for: Cellular Mechanical Phenotypes of Drought-Resistant and Drought-Sensitive Rice Species Distinguished by Double-Resonator Piezoelectric Cytometry Biosensors
Source: Biosensors (Basel). 2025 May 23;15(6):334. doi: 10.3390/bios15060334 (PMC12191338; doi:10.3390/bios15060334)
Supplement: Supplementary file 1 [file biosensors-15-00334-s001.zip › biosensors-3602761-supplementary.pdf]

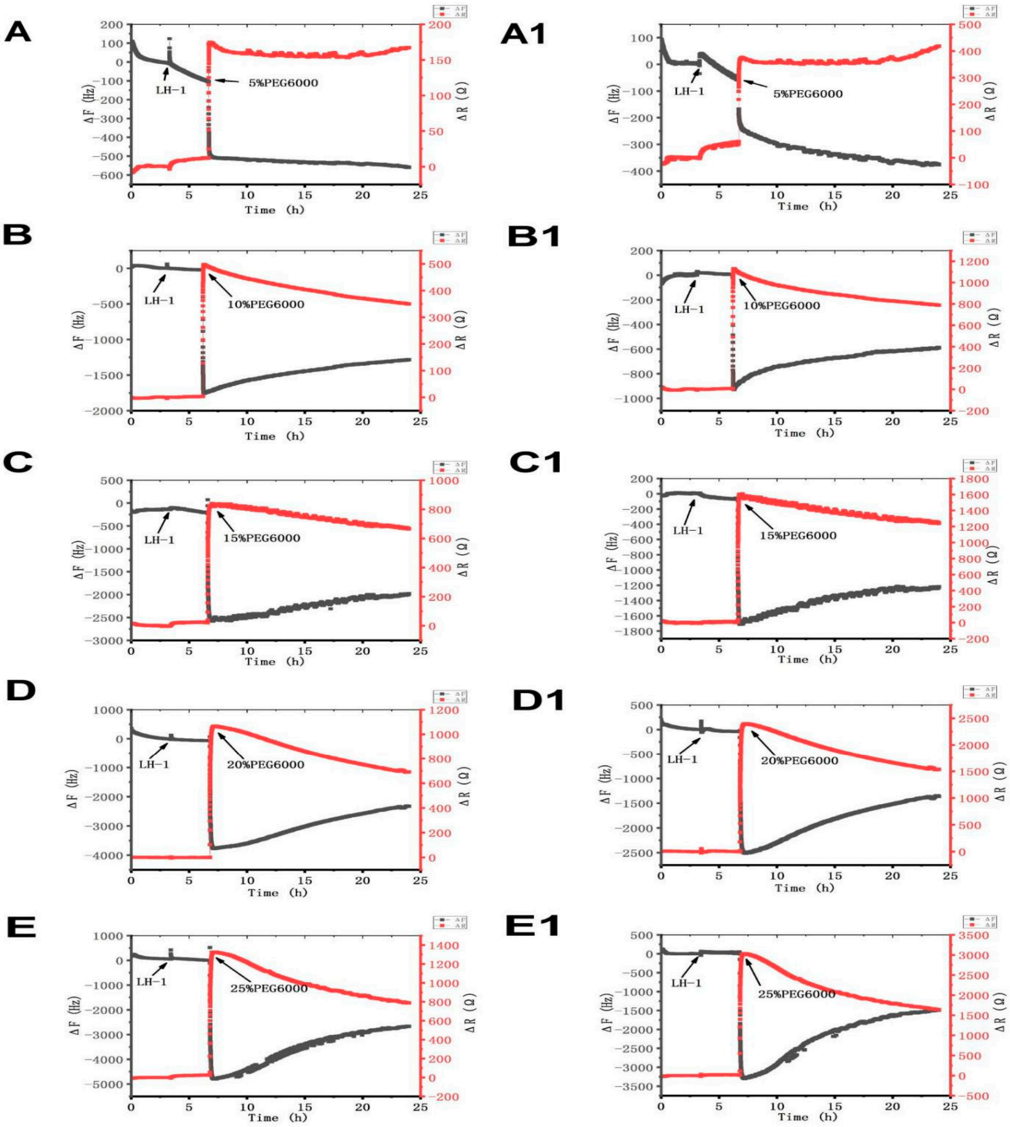


**Figure S1.** Changes in frequency and motional resistance of 9 MHz AT and BT cut chips during the adhesions of Lvhan No.1 rice cells followed by the treatments of different concentrations of PEG6000 stresses. (A, B, C, D, E): AT cut, (A1, B1, C1, D1, E1): BT cut, (A, A1): 5% PEG6000, (B, B1): 10%PEG6000, (C, C1) 15%PEG6000, (D, D1): 20%PEG6000, (E, E1): 25% PEG6000.

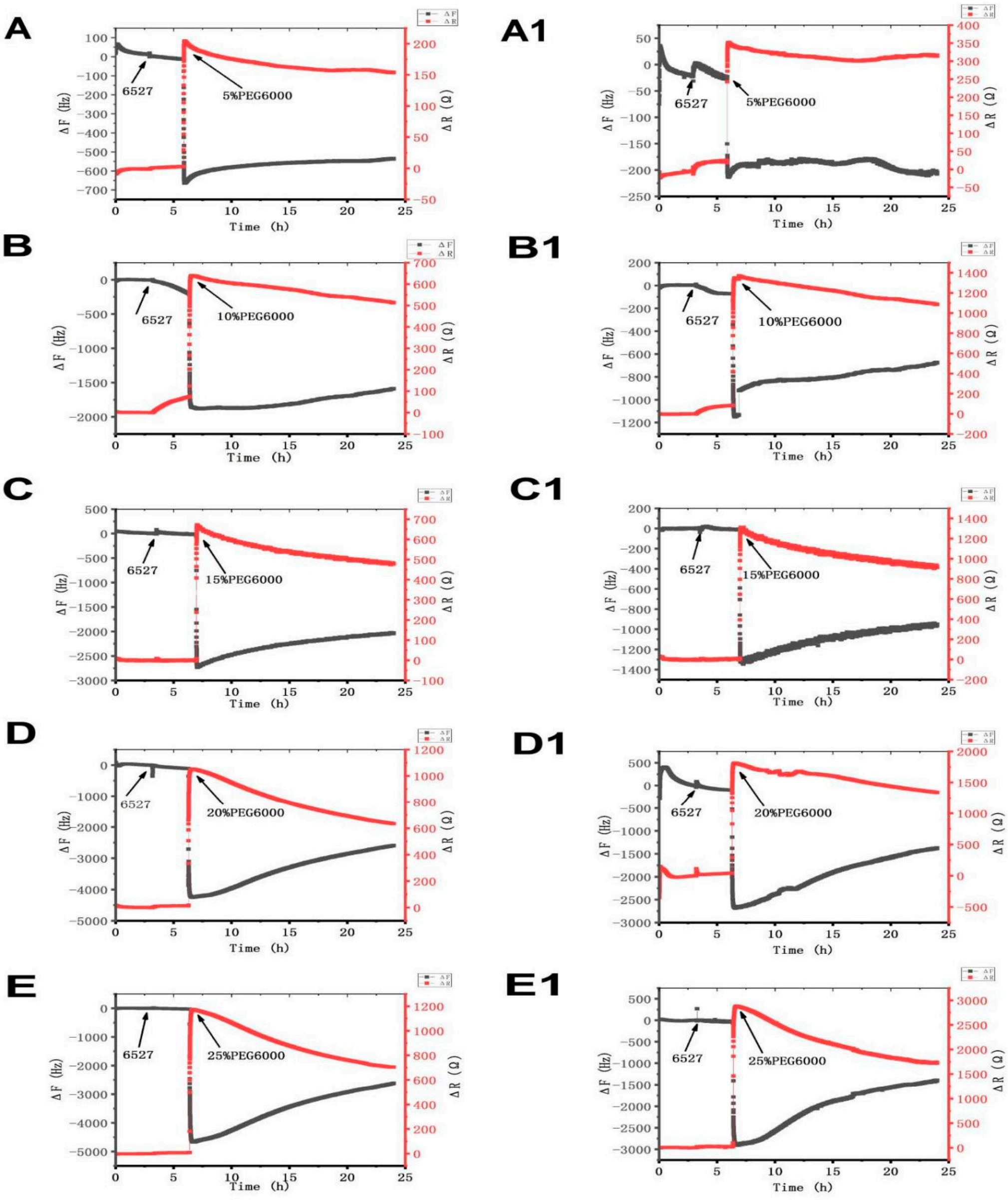


**Figure S2.** Changes in frequency and motional resistance of 9 MHz AT and BT cut chips during the adhesions of 6527 rice cells followed by the treatments of different concentrations of PEG6000 stresses. (A, B, C, D, E): AT cut, (A1, B1, C1, D1, E1): BT cut, (A, A1): 5% PEG6000, (B, B1): 10%PEG6000, (C, C1) 15%PEG6000, (D, D1): 20%PEG6000, (E, E1): 25% PEG6000.
